# 1-[2-(Di­methyl­aza­nium­yl)eth­yl]-1*H*-1,2,3,4-tetra­zole-5-thiol­ate

**DOI:** 10.1107/S1600536814001573

**Published:** 2014-01-29

**Authors:** Sadasivam Sharmila Tagore, Sivaraman Krishna, Sundaramoorthy Gomathi, Velusamy Sethuraman

**Affiliations:** a Periyar Maniammai University, Thanjavur 613 403, Tamil Nadu, India; b School of Chemistry, Bharathidasan University, Tiruchirappalli 620 024, Tamil Nadu, India

## Abstract

In the crystal structure of the title zwitterion, C_5_H_11_N_5_S, mol­ecules are linked *via* N—H⋯N hydrogen bonds, forming zigzag chains propagating along [010]. The chains are linked by C—H⋯S hydrogen bonds, forming two dimensional networks lying parallel to (001).

## Related literature   

For the biological activity of tetra­zoles, see: Juby *et al.* (1982 ▶); Tamilselvi & Mugesh (2009 ▶, 2011 ▶). For the general existence of zwitterions in other mol­ecules and the involvement of a protonated N atom in hydrogen bonding, see: Ruanwas *et al.* (2012 ▶); Ha (2012 ▶). For the biological activity of cefotiam (systematic name: (6*R*,7*R*)-7-{[2-(2-amino-1,3-thia­zol-4-yl)acet­yl]amino}-3-{[1-(2-di­methyl­amino­eth­yl)tetra­zol-5-yl), an anti­obiotic with DMETT [1-(2-(di­methyl­amino)-eth­yl)-1*H*-tetra­zole-5-thione] as a side chain, against enterobacteriacea, see: Garcia-Rodriguez *et al.* (1995 ▶); Polis & Tuazon (1985 ▶).
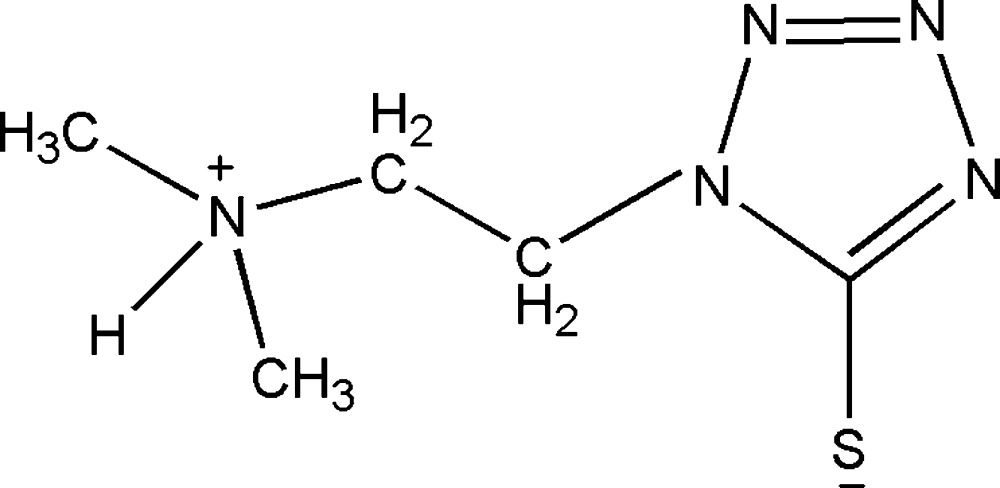


## Experimental   

### 

#### Crystal data  

C_5_H_11_N_5_S
*M* *_r_* = 173.26
Monoclinic, 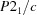

*a* = 7.1021 (1) Å
*b* = 11.2476 (2) Å
*c* = 10.7045 (2) Å
β = 102.479 (1)°
*V* = 834.89 (2) Å^3^
*Z* = 4
Mo *K*α radiation
μ = 0.33 mm^−1^
*T* = 100 K
0.41 × 0.21 × 0.14 mm

#### Data collection  

Bruker Kappa APEXII CCD diffractometer
Absorption correction: multi-scan (*SADABS*; Bruker, 2004 ▶) *T*_min_ = 0.876, *T*_max_ = 0.955
11518 measured reflections
3004 independent reflections
2618 reflections with *I* > 2σ(*I*)
*R*_int_ = 0.027

#### Refinement  

*R*[*F* ^2^ > 2σ(*F* ^2^)] = 0.038
*wR*(*F* ^2^) = 0.092
*S* = 1.05
3004 reflections
106 parameters
H atoms treated by a mixture of independent and constrained refinement
Δρ_max_ = 0.48 e Å^−3^
Δρ_min_ = −0.30 e Å^−3^

### 

Data collection: *APEX2* (Bruker, 2004 ▶); cell refinement: *SAINT* (Bruker, 2004 ▶); data reduction: *SAINT*; program(s) used to solve structure: *SHELXS97* (Sheldrick, 2008 ▶); program(s) used to refine structure: *SHELXL97* (Sheldrick, 2008 ▶); molecular graphics: *PLATON* (Spek, 2009 ▶), *Mercury* (Macrae *et al.*, 2008 ▶) and *POV-RAY* (Cason, 2004 ▶); software used to prepare material for publication: *PLATON* and *publCIF* (Westrip, 2010 ▶).

## Supplementary Material

Crystal structure: contains datablock(s) global, I. DOI: 10.1107/S1600536814001573/bv2228sup1.cif

Structure factors: contains datablock(s) I. DOI: 10.1107/S1600536814001573/bv2228Isup2.hkl

Supporting information file. DOI: 10.1107/S1600536814001573/bv2228Isup3.cml

CCDC reference:

Additional supporting information:  crystallographic information; 3D view; checkCIF report

## Figures and Tables

**Table 1:** Hydrogen-bond geometry (Å, °)

*D*—H⋯*A*	*D*—H	H⋯*A*	*D*⋯*A*	*D*—H⋯*A*
N5—H5⋯N4^i^	0.934 (16)	1.889 (17)	2.8054 (14)	166.2 (16)
C4—H4*C*⋯S1^ii^	0.98	2.83	3.7275 (13)	153

Symmetry codes: (i) 
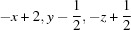
; (ii) 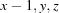.

**Fig. 1.**
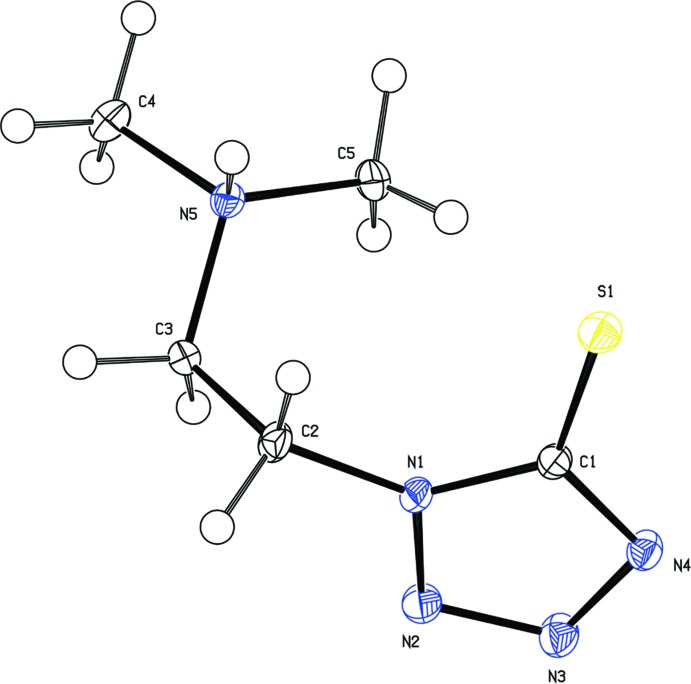
: The asymmetric unit of (I), shown in 50% probability displacement ellipsoids.

**Fig. 2.**
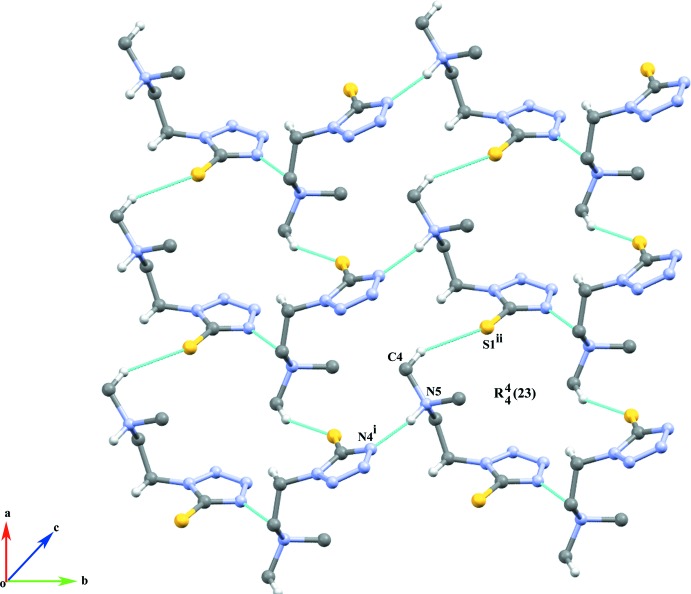
: A view of two dimension network formed by N—H···N and C—H···S hydrogen bonds. [symmetry code: (i) 2 - x, -1/2 + y, 1/2 - z; (ii) -1 + x, y, z].

## supplementary crystallographic information

### 1. Comment

A large number of tetrazole derivatives exhibit diverse pharmacological properties (Juby *et al.*, 1982) and some antibiotics with heterocyclic thiol side chains possess antithyroid activity (Tamilselvi & Mugesh, 2009). 1-(2-(Dimethylamino)-ethyl)-1*H*-tetrazole-5-thione (DMETT) exhibits thiol-thione tautomerism and thiolate anion formation which plays a vital role in the inhibition of the metallo-β-lactamases catalysed hydrolysis of cephalosporins (Tamilselvi & Mugesh, 2011). Cefotiam, a second-generation cephalosporin antibiotic, has a derivative of DMETT as a side chain which is more active against many of the Enterobacteriacea including Enterobacter, E·Coli, Klebsiella, Salmonella and Indole-positive Proteus species (Garcia-Rodriguez *et al.* 1995; Polis & Tuazon, 1985).

The current investigation focusses on the supramolecular hydrogen bonded patterns exhibited by the title compound (Scheme. 1).

The title compound (I), 1-[2-(dimethylazaniumyl)ethyl]-1*H*-1,2,3,4-tetrazole-5-thiolate tetrazole-5-thiolate (DMATT) crystallizes with one molecule in the asymmetric unit and it exists as a zwitterion with the positive charge on the ammonium nitrogen atom and the negative charge on the thiolate sulfur atom (Fig 1).

This compound exists as a zwitterion like many other amino compounds reported in the literature (Ruanwas, *et al.*, 2012; Ha, 2012). There is an intramolecular exchange of thiol proton to amino nitrogen and thus thiolate formation, which leads to the generation of a nucleophilic sulfur species (responsible for the inhibition activity). The geometry of the *tert*- ammonium group is found to be tetrahedral with bond angles ranging from 106.9 (11)–113.32 (9)°. The maximum deviation of the side chain at N1 position from the mean plane of tetrazole ring moiety is found to be 0.313 (10) Å. The molecular confirmation is inferred from the torsion angles N1—C2—C3—N5, C2—C3—N5—C4 and C2—C3—N5—C5 and the values are found to be -81.52 (12)°, -166.23 (9)°, and 70.26 (12)° respectively.

Two inversion related zwitterions present in the same plane are linked by a strong N—H···N hydrogen bond involving *tert*-ammonium group and ring N4^i^ atom [symmetry code: -*x* + 2, *y* - 1/2, -*z* + 1/2] (Table 1). This interaction extends along *b* axis leading to the generation of a wave like supramolecular chain. The supramolecular chains in the adjacent planes are connected *via* a weak C—H···S hydrogen bond. This hydrogen bond links one of the methyl C—H groups and S1^ii^ atom [symmetry code: *x* - 1, *y*, *z*] and generates another wave like supramolecular chain extending along *a* axis. The centrosymmetric N—H···N linkage between two zwitterions facilitates the effective occurrence of C—H···S hydrogen bond thus forming a ring motif with a graph set of *R*_4_^4^(23). This motif exists in both *a* and *b* axis and generates a two dimensional network as shown in Fig. 2.

### 2. Experimental

The title compound was obtained by dissolving DMETT (Sigma-Aldrich; 43.3mg, 0.25 mmol) in 20 ml of hot methanol, warming the resultant solution over a water bath for half an hour and then keeping it at room temperature for crystallization. After a week's time, colorless prismatic crystals were obtained

### 3. Refinement

The hydrogen atoms for the ammonium group (NH_4_^+^) was located in a difference Fourier map and refined freely. All other hydrogen atoms were positioned geometrically (C—H = 0.98–0.99 A) and were refined using a riding model, with *U*_iso_(H) = 1.2Ueq(C) for CH_2_ or 1.5Ueq(C) for CH_3_.

### Crystal data

**Table:**

C_5_H_11_N_5_S	*F*(000) = 368
*M**_r_* = 173.26	*D*_x_ = 1.378 Mg m^−^^3^
Monoclinic, *P*2_1_/*c*	Mo *K*α radiation, λ = 0.71073 Å
Hall symbol: -P 2ybc	Cell parameters from 3004 reflections
*a* = 7.1021 (1) Å	θ = 2.7–32.7°
*b* = 11.2476 (2) Å	µ = 0.33 mm^−^^1^
*c* = 10.7045 (2) Å	*T* = 100 K
β = 102.479 (1)°	Prism, colourless
*V* = 834.89 (2) Å^3^	0.41 × 0.21 × 0.14 mm
*Z* = 4	

### Data collection

**Table:**

Bruker Kappa APEXII CCD diffractometer	3004 independent reflections
Radiation source: fine-focus sealed tube	2618 reflections with *I* > 2σ(*I*)
Graphite monochromator	*R*_int_ = 0.027
ω and φ scan	θ_max_ = 32.7°, θ_min_ = 2.7°
Absorption correction: multi-scan (*SADABS*; Bruker, 2004)	*h* = −10→10
*T*_min_ = 0.876, *T*_max_ = 0.955	*k* = −16→16
11518 measured reflections	*l* = −16→16

### Refinement

**Table:**

Refinement on *F*^2^	Primary atom site location: structure-invariant direct methods
Least-squares matrix: full	Secondary atom site location: difference Fourier map
*R*[*F*^2^ > 2σ(*F*^2^)] = 0.038	Hydrogen site location: inferred from neighbouring sites
*wR*(*F*^2^) = 0.092	H atoms treated by a mixture of independent and constrained refinement
*S* = 1.05	*w* = 1/[σ^2^(*F*_o_^2^) + (0.0391*P*)^2^ + 0.4372*P*] where *P* = (*F*_o_^2^ + 2*F*_c_^2^)/3
3004 reflections	(Δ/σ)_max_ = 0.002
106 parameters	Δρ_max_ = 0.48 e Å^−^^3^
0 restraints	Δρ_min_ = −0.30 e Å^−^^3^

### Special details

**Table:**

Geometry. Bond distances, angles *etc*. have been calculated using the rounded fractional coordinates. All su's are estimated from the variances of the (full) variance-covariance matrix. The cell e.s.d.'s are taken into account in the estimation of distances, angles and torsion angles
Refinement. Refinement on *F*^2^ for ALL reflections except those flagged by the user for potential systematic errors. Weighted *R*-factors *wR* and all goodnesses of fit *S* are based on *F*^2^, conventional *R*-factors *R* are based on *F*, with *F* set to zero for negative *F*^2^. The observed criterion of *F*^2^ > σ(*F*^2^) is used only for calculating -*R*-factor-obs *etc*. and is not relevant to the choice of reflections for refinement. *R*-factors based on *F*^2^ are statistically about twice as large as those based on *F*, and *R*-factors based on ALL data will be even larger.

### Fractional atomic coordinates and isotropic or equivalent isotropic displacement parameters (Å^2^)

**Table:**

	*x*	*y*	*z*	*U*_iso_*/*U*_eq_	
S1	1.21899 (4)	1.07521 (3)	0.25401 (3)	0.0158 (1)	
N1	0.85227 (13)	1.05977 (8)	0.10942 (9)	0.0111 (2)	
N2	0.71652 (14)	1.12926 (9)	0.03430 (9)	0.0146 (3)	
N3	0.79116 (14)	1.23401 (9)	0.03491 (10)	0.0155 (3)	
N4	0.97380 (13)	1.23578 (9)	0.10896 (9)	0.0129 (2)	
N5	0.72606 (14)	0.90248 (8)	0.33382 (9)	0.0109 (2)	
C1	1.01325 (15)	1.12532 (10)	0.15599 (10)	0.0112 (3)	
C2	0.81956 (17)	0.93255 (10)	0.12032 (11)	0.0133 (3)	
C3	0.66221 (16)	0.90062 (10)	0.19086 (10)	0.0120 (3)	
C4	0.57703 (17)	0.84181 (11)	0.39090 (11)	0.0151 (3)	
C5	0.76474 (17)	1.02474 (10)	0.38754 (10)	0.0137 (3)	
H2A	0.94160	0.89510	0.16520	0.0160*	
H2B	0.78540	0.89820	0.03320	0.0160*	
H3A	0.55390	0.95710	0.16510	0.0140*	
H3B	0.61320	0.82020	0.16390	0.0140*	
H4A	0.61680	0.84390	0.48440	0.0230*	
H4B	0.56360	0.75900	0.36190	0.0230*	
H4C	0.45320	0.88280	0.36380	0.0230*	
H5	0.839 (2)	0.8576 (16)	0.3566 (16)	0.024 (4)*	
H5A	0.65260	1.07550	0.35540	0.0210*	
H5B	0.87790	1.05780	0.36130	0.0210*	
H5C	0.78910	1.02120	0.48110	0.0210*	

### Atomic displacement parameters (Å^2^)

**Table:**

	*U*^11^	*U*^22^	*U*^33^	*U*^12^	*U*^13^	*U*^23^
S1	0.0125 (1)	0.0164 (2)	0.0171 (1)	0.0012 (1)	0.0001 (1)	0.0039 (1)
N1	0.0124 (4)	0.0083 (4)	0.0120 (4)	−0.0003 (3)	0.0014 (3)	0.0014 (3)
N2	0.0142 (4)	0.0123 (5)	0.0155 (4)	0.0007 (4)	−0.0004 (3)	0.0024 (3)
N3	0.0141 (4)	0.0127 (5)	0.0186 (5)	0.0001 (4)	0.0009 (3)	0.0021 (3)
N4	0.0121 (4)	0.0106 (4)	0.0154 (4)	−0.0002 (3)	0.0020 (3)	0.0014 (3)
N5	0.0122 (4)	0.0089 (4)	0.0115 (4)	0.0003 (3)	0.0025 (3)	0.0003 (3)
C1	0.0132 (4)	0.0099 (5)	0.0110 (4)	−0.0007 (4)	0.0039 (3)	0.0000 (3)
C2	0.0185 (5)	0.0080 (5)	0.0144 (5)	−0.0018 (4)	0.0056 (4)	−0.0008 (4)
C3	0.0143 (5)	0.0108 (5)	0.0101 (4)	−0.0027 (4)	0.0012 (3)	−0.0006 (3)
C4	0.0162 (5)	0.0139 (5)	0.0166 (5)	−0.0007 (4)	0.0065 (4)	0.0028 (4)
C5	0.0170 (5)	0.0101 (5)	0.0137 (5)	−0.0014 (4)	0.0024 (4)	−0.0026 (4)

### Geometric parameters (Å, º)

**Table:**

S1—C1	1.7003 (11)	C2—C3	1.5207 (17)
N1—N2	1.3609 (14)	C2—H2A	0.9900
N1—C1	1.3612 (14)	C2—H2B	0.9900
N1—C2	1.4584 (14)	C3—H3A	0.9900
N2—N3	1.2914 (14)	C3—H3B	0.9900
N3—N4	1.3664 (14)	C4—H4A	0.9800
N4—C1	1.3471 (15)	C4—H4B	0.9800
N5—C3	1.4994 (14)	C4—H4C	0.9800
N5—C4	1.4962 (16)	C5—H5A	0.9800
N5—C5	1.4929 (14)	C5—H5B	0.9800
N5—H5	0.934 (16)	C5—H5C	0.9800
			
S1···H2A	2.8400	C5···N1	3.1945 (14)
S1···H3A^i^	3.0500	C5···N3^x^	3.1239 (15)
S1···H4C^i^	2.8300	C1···H5B	2.6900
S1···H5A^i^	3.0400	C1···H5^ii^	2.830 (18)
S1···H5B	2.9000	C1···H2B^iii^	2.7300
S1···H3B^ii^	3.0600	C2···H5B	2.8900
S1···H4B^ii^	3.0000	H2A···S1	2.8400
S1···H2B^iii^	3.0800	H2A···H5	2.3600
S1···H4A^iv^	2.9400	H2B···S1^iii^	3.0800
S1···H5C^iv^	3.0500	H2B···N4^iii^	2.9400
N1···N4	2.1601 (14)	H2B···C1^iii^	2.7300
N1···N5	3.2610 (13)	H3A···S1^xi^	3.0500
N1···C5	3.1945 (14)	H3A···N2	2.7800
N2···C4^v^	3.3800 (16)	H3A···H4C	2.5300
N2···N4	2.1867 (14)	H3A···H5A	2.4100
N2···C3^vi^	3.2180 (15)	H3A···N2^vi^	2.7200
N3···C5^vii^	3.1239 (15)	H3B···H4B	2.3300
N3···C4^v^	3.1363 (16)	H3B···S1^viii^	3.0600
N4···C4^ii^	3.4054 (16)	H3B···N2^vi^	2.8600
N4···N1	2.1601 (14)	H4A···H5C	2.3400
N4···N5^ii^	2.8054 (14)	H4A···S1^iv^	2.9400
N5···N1	3.2610 (13)	H4B···H3B	2.3300
N5···N4^viii^	2.8054 (14)	H4B···S1^viii^	3.0000
N1···H5B	2.6600	H4B···N2^ix^	2.8800
N2···H3A	2.7800	H4C···S1^xi^	2.8300
N2···H4B^v^	2.8800	H4C···H3A	2.5300
N2···H3A^vi^	2.7200	H4C···N3^ix^	2.7900
N2···H3B^vi^	2.8600	H5···H2A	2.3600
N3···H4C^v^	2.7900	H5···N4^viii^	1.889 (17)
N3···H5C^vii^	2.8100	H5···C1^viii^	2.830 (18)
N3···H5A^vii^	2.9000	H5A···S1^xi^	3.0400
N4···H2B^iii^	2.9400	H5A···H3A	2.4100
N4···H5^ii^	1.889 (17)	H5A···N3^x^	2.9000
C1···C5	3.5255 (16)	H5B···S1	2.9000
C1···C2^iii^	3.4785 (16)	H5B···N1	2.6600
C2···C1^iii^	3.4785 (16)	H5B···C1	2.6900
C3···N2^vi^	3.2180 (15)	H5B···C2	2.8900
C4···N3^ix^	3.1363 (16)	H5C···H4A	2.3400
C4···N2^ix^	3.3800 (16)	H5C···S1^iv^	3.0500
C4···N4^viii^	3.4054 (16)	H5C···N3^x^	2.8100
C5···C1	3.5255 (16)		
			
N2—N1—C1	109.70 (9)	C3—C2—H2B	109.00
N2—N1—C2	120.40 (9)	H2A—C2—H2B	108.00
C1—N1—C2	129.63 (10)	N5—C3—H3A	109.00
N1—N2—N3	106.46 (9)	N5—C3—H3B	109.00
N2—N3—N4	110.70 (9)	C2—C3—H3A	109.00
N3—N4—C1	107.34 (9)	C2—C3—H3B	109.00
C3—N5—C4	109.02 (9)	H3A—C3—H3B	108.00
C3—N5—C5	113.32 (8)	N5—C4—H4A	109.00
C4—N5—C5	110.51 (9)	N5—C4—H4B	109.00
C5—N5—H5	108.7 (11)	N5—C4—H4C	109.00
C3—N5—H5	108.2 (10)	H4A—C4—H4B	109.00
C4—N5—H5	106.9 (10)	H4A—C4—H4C	109.00
S1—C1—N4	128.17 (9)	H4B—C4—H4C	109.00
S1—C1—N1	126.03 (9)	N5—C5—H5A	109.00
N1—C1—N4	105.80 (9)	N5—C5—H5B	109.00
N1—C2—C3	114.73 (9)	N5—C5—H5C	109.00
N5—C3—C2	114.26 (9)	H5A—C5—H5B	109.00
N1—C2—H2A	109.00	H5A—C5—H5C	109.00
N1—C2—H2B	109.00	H5B—C5—H5C	109.00
C3—C2—H2A	109.00		
			
C1—N1—N2—N3	−0.17 (12)	N1—N2—N3—N4	−0.04 (12)
C2—N1—N2—N3	−174.75 (10)	N2—N3—N4—C1	0.24 (12)
N2—N1—C1—S1	179.98 (9)	N3—N4—C1—S1	−180.00 (8)
N2—N1—C1—N4	0.31 (12)	N3—N4—C1—N1	−0.33 (12)
C2—N1—C1—S1	−6.09 (17)	C4—N5—C3—C2	−166.23 (9)
C2—N1—C1—N4	174.24 (10)	C5—N5—C3—C2	70.26 (12)
N2—N1—C2—C3	−68.63 (13)	N1—C2—C3—N5	−81.52 (12)
C1—N1—C2—C3	118.00 (12)		

Symmetry codes: (i) *x*+1, *y*, *z*; (ii) −*x*+2, *y*+1/2, −*z*+1/2; (iii) −*x*+2, −*y*+2, −*z*; (iv) −*x*+2, −*y*+2, −*z*+1; (v) −*x*+1, *y*+1/2, −*z*+1/2; (vi) −*x*+1, −*y*+2, −*z*; (vii) *x*, −*y*+5/2, *z*−1/2; (viii) −*x*+2, *y*−1/2, −*z*+1/2; (ix) −*x*+1, *y*−1/2, −*z*+1/2; (x) *x*, −*y*+5/2, *z*+1/2; (xi) *x*−1, *y*, *z*.

### Hydrogen-bond geometry (Å, º)

**Table:**

*D*—H···*A*	*D*—H	H···*A*	*D*···*A*	*D*—H···*A*
N5—H5···N4^viii^	0.934 (16)	1.889 (17)	2.8054 (14)	166.2 (16)
C2—H2*A*···S1	0.99	2.84	3.3039 (12)	109
C4—H4*C*···S1^xi^	0.98	2.83	3.7275 (13)	153

Symmetry codes: (viii) −*x*+2, *y*−1/2, −*z*+1/2; (xi) *x*−1, *y*, *z*.
